# Network science for the identification of novel therapeutic targets in epilepsy

**DOI:** 10.12688/f1000research.8214.1

**Published:** 2016-05-16

**Authors:** Rod C. Scott

**Affiliations:** 1 Department of Neurological Sciences, University of Vermont, Burlington, VT, USA; 2 Neurosciences Unit, UCL Institute of Child Health, London, UK

**Keywords:** epilepsy, therapeutic targets, network science

## Abstract

The quality of life of children with epilepsy is a function of seizures and associated cognitive and behavioral comorbidities. Current treatments are not successful at stopping seizures in approximately 30% of patients despite the introduction of multiple new antiepileptic drugs over the last decade. In addition, modification of seizures has only a modest impact on the comorbidities. Therefore, novel approaches to identify therapeutic targets that improve seizures and comorbidities are urgently required. The potential of network science as applied to genetic, local neural network, and global brain data is reviewed. Several examples of possible new therapeutic approaches defined using novel network tools are highlighted. Further study to translate the findings into clinical practice is now required.

## Introduction

Children with epilepsy have seizures that are frequently associated with cognitive and behavioral impairments ^1^. In combination, these factors negatively impact quality of life ^2^ and greatly diminish the ability of children with epilepsy to develop into successful adults. It is therefore critical that treatments aimed at modifying adverse outcomes are developed. Current therapies are largely targeted toward seizures and include antiepileptic drugs and dietary and surgical therapies. Although these therapies are effective in the treatment of seizures, approximately 30% of patients continue to have seizures despite maximal therapy ^3^. Specific therapies for behavioral difficulties include stimulants ^4, 5^, antipsychotics ^6, 7^, and antidepressants ^6, 7^. Therapies for learning impairments are largely educational. In all of these cases, the therapy targets symptoms without modifying the brain disorder responsible for the seizures and the associated morbidities. In order to develop new therapies, it is important that mechanisms of the underlying brain disorders are understood so that therapies can be targeted towards modifying those abnormalities and thereby improve outcomes.

The mechanisms underpinning adverse outcomes can be studied at a variety of levels including the level of genes, receptors, cell signaling pathways, and networks. Network science is providing new ways of understanding disease mechanisms and could lead to novel therapies targeted *directly* at modifying network abnormalities underlying seizures and comorbidities, irrespective of the genetic or cell signaling abnormalities ^8^ ( Figure 1). This review will explore the potential of network science to contribute to the identification of novel therapeutic targets.

**Figure 1.**
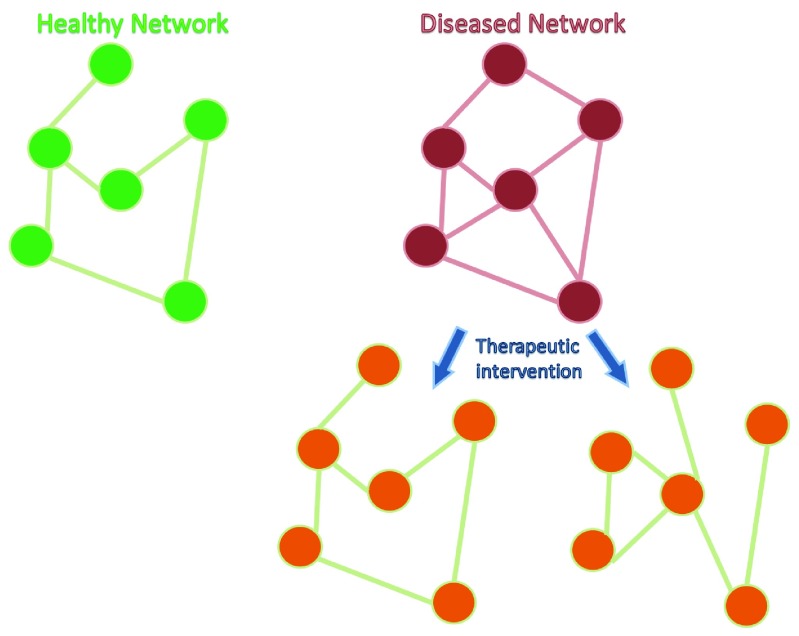
Cartoon representing healthy and diseased networks. Each node in the network could represent a gene, transcriptional module, single neuron, or brain region. The lines between the nodes represent relevant interactions between the nodes. Conceptually, the structure of the network is different in disease states compared to controls. In this example, the diseased network is over-connected (e.g. hypersynchrony in an epileptic brain). Targeting the network directly using drugs, brain stimulation, cell therapy, or transcranial magnetic stimulation approaches could modify the diseased network in order to allow more normal function. It remains unknown whether restoration of networks to normal (left network post-therapy) or modification to a network with similar phenotype to normal (right network post-therapy) is required. Understanding of these system-level mechanisms could lead to new treatments or optimization of currently used clinical tools, such as deep brain stimulation.

## Understanding disease mechanisms at network levels

Disease mechanisms in the context of epilepsy can be conceptualized in at least two ways. The first is encapsulated in the epileptic encephalopathy hypothesis, which states that epileptic activity causes cognitive and behavioral impairments over and above those expected for the underlying etiology ^9^. In this circumstance, it can be argued that epileptic activity itself modifies neural networks in a way that no longer supports normal cognitive and behavioral function. The therapeutic implication is that treatment of seizures and interictal discharges will positively modify adverse outcomes. Despite many years of trying to improve cognitive and behavioral outcomes by targeting seizures, the positive impacts are rather modest ^1, 10, 11^. A common suggestion is that the treatment of seizures prevents any further decline in abilities. If correct, this suggests that alternative approaches to *improving* outcomes are required.

The second concept places etiology in the forefront. In this view, patients with epilepsy all have an underlying etiology (even if it is unrecognized) and this is directly responsible for the neural network disruptions that lead to the symptoms of seizures and adverse cognitive and behavioral outcomes. It remains possible that seizures also make a contribution to the modification of networks, although it is difficult to clearly identify the magnitude of the contribution ^12, 13^. However, if etiology is the major contributor, then treatment of seizures is unlikely to have major positive impact, as observed in clinical practice. If seizures and cognitive/behavioral impairments are primarily a function of etiology, then disease-modifying therapies would be expected to improve all of the symptoms. Although this is an area receiving increasing attention, no such therapies are yet available in clinical practice.

The application of network science to these issues is gaining popularity ^8^. Network science is a mixture of graph theory, dynamical systems theory, and ideas from statistical mechanics ^8^. These tools can be applied to genetic, electrophysiological, and magnetic resonance data. The appropriate application of network science to epilepsy requires an interdisciplinary approach involving clinicians, translational neuroscientists, computer scientists, and mathematicians. Examples of novel insights that are arising from these interdisciplinary collaborations are discussed below.

## Gene regulation networks

Genetic approaches have made significant contributions to the understanding of neurological disorders associated with seizures ^14– 17^. The hope is that gene replacement therapy may restore function, as in an animal model of Rett syndrome ^18^. This approach relates to single gene disorders, and the list of genes associated with epilepsies is rapidly growing ^17, 19^. Although it may be possible to replace the identified genes, many abnormalities are extremely rare, raising an important concern about the practical use of such approaches. An alternative approach is to use systems genetics to characterize the genetic regulation of pathophysiological pathways. This approach has previously been used to explore the pathophysiology of type 1 diabetes ^20^, autism ^21, 22^, febrile convulsions ^23– 25^, and the latter’s association with mesial temporal lobe epilepsy ^24^. An example of how systems genetics could identify a therapeutic target in epilepsy comes from a study of temporal lobe epilepsy ^26^. Johnson *et al*. evaluated gene expression networks in post-surgical hippocampal tissue from patients with temporal lobe epilepsy. They identified a specialized, highly expressed transcriptional module encoding proconvulsive cytokine and Toll-like receptor signaling genes. Sestrin 3 positively regulates this module in macrophages, microglia, and neurons. This finding is also present in pilocarpine-exposed mice, which demonstrated pilocarpine-induced status epilepticus with subsequent temporal lobe epilepsy, giving further credibility to the clinical finding. Importantly, knockdown of Sestrin 3 in zebrafish attenuates chemically induced behavioral seizures. Therefore, it is possible that modulation of Sestrin 3 could modulate seizures in humans. It is unlikely that this target would have been identified without the mathematical approaches inherent in systems genetics. This study used tissue from epilepsy surgery, was carried out in adults, and targeted an etiology common in adults. However, the pathophysiological processes leading to mesial temporal sclerosis (MTS) frequently occur during development. Children with epilepsies frequently have malformations of cortical development, and similar approaches could be applied to this surgical tissue and have potential to identify other novel therapeutic targets. Modification of genetic signaling can be achieved with small interfering RNA (siRNA) and microRNA (miRNA) approaches. Identification of specific genes or regulatory gene networks could provide critical targets for these approaches that are already being tested in the clinical domain ^27^. These approaches can also be applied to epigenetic data and could provide important insights in that domain as well.

## Local neural networks

Another level at which network approaches may identify therapeutic targets is at the level of local neuronal networks. This requires the identification of system-level mechanisms at the level of changes in neural circuitry that directly impair the ability of that circuit to support cognitive behavior. Information processing in a neural system is a function of the rate of firing of neurons (rate coding) ^28– 32^, the precise timing of action potential firing with respect to oscillatory activity (temporal coding) ^33, 34^, and joint activation of ensembles of neurons (population coding) ^35– 38^. It is known that each of these components of information processing can be abnormal in a variety of animal models of epilepsies. Rate coding as described by hippocampal place cells is disrupted following early life seizures and in the context of MTS ^39– 42^. The spatial fidelity of these cells is reduced and this is consistent with the abnormalities in spatial cognition identified in these models ^41, 43– 45^. Temporal and population coding have also been evaluated in models of MTS and found to be abnormal ^36, 39^. For example, excessive synchrony of CA1 pyramidal cell firing predicts poor performance in a spatial alternation task, suggesting that the organization of neuronal firing within a neural population is important for behavior ^36^. It remains uncertain whether this excessive synchrony is important for seizure generation, but modulation of this synchrony could both reduce seizures and improve cognition.

Many of the epilepsy models described above have associated structural abnormalities. Remarkably, cognitive outcomes can be improved with environmental enrichment and overtraining strategies, even in the context of the structural brain abnormalities ^46^, suggesting that structure-function relationships are not fixed. This could be exploited for therapeutic advantage if the network-level changes associated with these improvements could be identified. This remains an area of active research. However, some system-level interventions have been shown to improve outcomes in epilepsy. In humans, stimulation devices are known to reduce seizures and there is some evidence that stimulation of the entorhinal cortex can improve cognition ^47^. A detailed understanding of how networks need to be modified to improve outcomes would allow optimization of these tools. Electrical stimulation has the potential downside of activating all cell types, which may limit the possibility of more subtly altering neuronal interactions. In the future, optogenetic stimulation may allow more precise modulation of neural network behavior by activating only certain cell types.

Interneurons are critical for the organization of pyramidal cells ^48^ and therefore may represent a cell target in epilepsy. Implantation of interneuron precursors into animal models of MTS reduces seizures ^49– 51^ and, at least in some experiments, improves cognitive outcomes ^49^. In addition, optogenetic stimulation of interneurons using a closed loop system also reduces seizures ^52^. The system-level mechanisms underlying the improvements have not yet been explored in these models, but such studies could ultimately provide essential information for optimizing parameters and maximizing outcomes.

Although stimulation approaches are the most obvious application of the sort of system-level information described above, drug interventions may also be possible. For example, adrenocorticotropic hormone (ACTH) administration to rodents exposed to early life seizures improves attentional outcomes despite having no effect on the seizures ^53^. This suggests that ACTH is modulating information processing in a way that is currently unknown but deserves future study. The implication for the treatment of patients is that medications that do not influence seizures but improve cognitive outcomes may be beneficial. Therefore, outcome measures for clinical trials may need to include cognitive outcomes, such as in the UKISS trial of infantile spasms ^54^, and may even have to be designed to have cognition as the main outcome measure. Another speculative drug approach is the use of Designer Receptors Exclusively Activated by Designer Drugs (DREADDS) ^52^. This approach may also allow targeting of certain cell types within a network in order to modify the population activity of that network.

## Global networks

The study of global networks within the context of epilepsy has used magnetic resonance, electroencephalography, and magnetoencephalography approaches. Although there is a wealth of data on the phenomenology of connectivity (see for example 55– 59), there are far less data on how this information can be used for therapeutic purposes. However, an understanding of distributed networks could identify regions of the brain that are critical for seizure generation or for any relevant cognitive function, even if that area of the brain is not the obviously abnormal piece of tissue. An excellent example of this is from a study of patients with periventricular nodular heterotopia associated with seizures ^60^. Functional MRI was used to identify aberrant functional connectivity between the heterotopia and normal-looking cortex. Abnormal connectivity was identified between the heterotopia and areas of the cortex that were not consistent across patients. Transcranial magnetic stimulation (TMS) was used to show that the connected cortical areas were hyperexcitable, suggesting that the authors had identified an epileptogenic network. This raises the possibility that TMS targeted at the cortical site identified using network approaches could reduce seizures in patients with deep-seated epileptogenic foci.

Another potential use of network approaches is in epilepsy surgery. Despite state-of-the-art selection methods, investigations for identifying the epileptogenic focus, and excellent surgical technique, many patients with epilepsy fail to become seizure free. In these patients, it is possible that the epileptogenic networks have not been adequately disrupted by surgery and therefore a more detailed understanding of the network could lead to tailored resections based on that information. A variety of network measures have been used to describe the distributed abnormal networks in patients with focal epilepsy, particularly those with MTS ^55, 61– 63^. Patients who subsequently failed to become seizure free often had different network changes to those patients who did become seizure free. The next challenge is to establish how that information can be used to maximize surgical outcomes.

## Conclusion

The goal of this article was not to provide an exhaustive review of network science as applied to epilepsy but rather to provide examples of how these methods have enormous potential to contribute to the treatment of patients with epilepsy. The overriding concept is that an understanding of networks from genetic, local neural, and global brain data can identify new therapeutic targets for both seizures and associated comorbidities. Modification of these targets can be with drugs, stimulation devices, surgical approaches, and TMS, ultimately maximizing the quality of life of patients with epilepsy.
